# Retrospective Analysis of the Predictive Value of ^18^F-FDG PET/CT Metabolic Parameters for PD-L1 Expression in Cervical Cancer

**DOI:** 10.3390/diagnostics13061015

**Published:** 2023-03-07

**Authors:** Jianfeng Ji, Weiqiang Pang, Jinling Song, Xiawan Wang, Huarong Tang, Yunying Liu, Heqing Yi, Yun Wang, Qing Gu, Linfa Li

**Affiliations:** 1 Department of Nuclear Medicine, Zhejiang Cancer Hospital, Institute of Basic Medicine and Cancer (IBMC), Chinese Academy of Sciences, Hangzhou 310022, China; 2 Key Laboratory of Head & Neck Cancer Translational Research of Zhejiang Province, Hangzhou 310022, China; 3 Department of Radiation Oncology, Zhejiang Cancer Hospital, Institute of Basic Medicine and Cancer (IBMC), Chinese Academy of Sciences, Hangzhou 310022, China; 4 Department of Pathology, Zhejiang Cancer Hospital, Institute of Basic Medicine and Cancer (IBMC), Chinese Academy of Sciences, Hangzhou 310022, China

**Keywords:** ^18^F-FDG PET/CT, PD-L1, cervical cancer

## Abstract

Background: Immunotherapy targeting PD-1/PD-L1 has been proven to be effective for cervical cancer treatment. To explore non-invasive examinations for assessing the PD-L1 status in cervical cancer, we performed a retrospective study to investigate the predictive value of ^18^F-FDG PET/CT. Methods: The correlations between PD-L1 expression, clinicopathological characteristics and ^18^F-FDG PET/CT metabolic parameters were evaluated in 74 cervical cancer patients. The clinicopathological characteristics included age, histologic type, tumor differentiation, FIGO stage and tumor size. The metabolic parameters included maximum standard uptake (SUVmax), mean standard uptake (SUVmean), total lesion glycolysis (TLG) and tumor metabolic volume (MTV). Results: In univariate analysis, SUVmax, SUVmean, TLG, tumor size and tumor differentiation were obviously associated with PD-L1 status. SUVmax (rs = 0.42) and SUVmean (rs = 0.40) were moderately positively correlated with the combined positive score (CPS) for PD-L1 in Spearman correlation analysis. The results of multivariable analysis showed that the higher SUVmax (odds ratio = 2.849) and the lower degree of differentiation (Odds Ratio = 0.168), the greater probability of being PD-L1 positive. The ROC curve analysis demonstrated that when the cut-off values of SUVmax, SUVmean and TLG were 10.45, 6.75 and 143.4, respectively, the highest accuracy for predicting PD-L1 expression was 77.0%, 71.6% and 62.2%, respectively. The comprehensive predictive ability of PD-L1 expression, assessed by combining SUVmax with tumor differentiation, showed that the PD-L1-negative rate was 100% in the low probability group, whereas the PD-L1-positive rate was 84.6% in the high probability group. In addition, we also found that the H-score of HIF-1α was moderately positively correlated with PD-L1 CPS (rs = 0.51). Conclusions: The SUVmax and differentiation of the primary lesion were the optimum predictors for PD-L1 expression in cervical cancer. There was a great potential for ^18^F-FDG PET/CT in predicting PD-L1 status and selecting cervical cancer candidates for PD1/PD-L1 immune checkpoint therapy.

## 1. Introduction

In the world, the morbidity and tumor-related mortality of cervical cancer both rank fourth in all malignant tumors among women, with an incidence of roughly 604,000 new cases and more than three hundred thousand deaths annually [1]. With the promotion of HPV vaccines and the development of cancer screening technology, the incidence rate of cervical cancer has been significantly reduced. However, there are limited treatment options and serious side effects and most patients are diagnosed at an advanced stage [2]. The routine management of recurrent or metastatic cervical cancer has been an intractable clinical problem as a result of the insufficiency of existing treatment methods [3].

Immune checkpoints (ICs) are recognized to be a series of immunosuppressive molecules expressed on immune cells that have an inhibitory effect on immune activation and normally play a critical role in autoimmune prevention. In pathological conditions, the overexpression or hyperfunction of ICs can lead to immunity inhibition and contribute to tumorigenesis. Immune checkpoint blockades (ICBs), especially the inhibitors targeted to programmed cell death-1 (PD-1) or programmed death-ligand 1 (PD-L1), which can release the brakes of the hosts’ immune system to achieve durable clinical responses, have resulted in a transformation of cancer treatment [4]. Recently, the PD-1/PD-L1 inhibitors have become recommended treatments for cervical cancer patients in advanced, recurrent and/or metastatic conditions [5]. The research data from Keynote-826 (NCT03635567) in 2021 pointed out that [6] compared with chemotherapy ± bevacizumab, pembrolizumab combined with chemotherapy ± bevacizumab led to a 36% reduction in the death risk and significantly prolonged progression-free survival (PFS) and overall survival (OS) in patients scoring ≥ 1 on the combined positive score (CPS) for PD-L1 expression. Based on this, pembrolizumab (Keytruda, Merck & Co., Kenilworth, NJ, USA) has been approved by the US Food and Drug Administration (FDA) for combination therapy with chemotherapeutics in persistent, recurrent or metastatic cervical cancer with PD-L1 CPS ≥ 1. Tumors with higher expression of PD-L1 may exhibit better responses from immune checkpoint inhibitors.

Immunohistochemistry (IHC) is currently the primary method for quantifying PD-L1. The tumor tissues of cervical cancer patients are always obtained by invasive methods, such as puncture biopsy or surgical resection. However, it is always difficult for patients with recurrence and metastasis to obtain tumor tissues. 2-Deoxy-2-[^18^F] fluoro-D-glucose (^18^F-FDG) positron emission tomography/computed tomography (PET/CT) is acknowledged as an important non-invasive whole-body molecular imaging modality for evaluation of various tumors [7,8,9,10,11,12]. It has been reported that ^18^F-FDG PET/CT could be used to predict multiple molecular phenotypes for malignant tumors, for instance, HER2 expression in gastric cancer [13], EGFR mutations and positive ALK expression in NSCLC [14]. It has also been confirmed that high glucose metabolism was associated with tumor PD-L1 expression in various types of cancers [15,16,17].

In previous studies, research focusing on the relationship between the metabolic parameters of ^18^F-FDG PET/CT and the expression levels of PD-L1 in cervical cancer has not been reported. Thus, a retrospective study was performed by us to investigate the relevance of FDG PET/CT glucose metabolism to PD-L1 expression and evaluate the predictive value in cervical cancer.

## 2. Materials and Methods

### 2.1. Patients

A total of 74 cervical cancer patients who had received examination by ^18^F-FDG PET/CT preoperatively at Zhejiang Cancer Hospital from December 2016 to August 2021 were retrospectively collected. The inclusion criteria are as follows: (1) all cervical cancer cases were confirmed by postoperative histopathology; (2) there was no history of malignant tumor or other simultaneously suffered primary tumor; and (3) no prior systemic or local treatment had been performed before examination. This retrospective study was approved by Medical Ethics Committee of Zhejiang Cancer Hospital (IRB-2020-330) and the requirement of informed patient consent was waived.

### 2.2. ^18^F-FDG PET/CT Protocol and Image Interpretation

Before the tracer injection, all patients were required to fast for 4–6 h to ensure venous blood glucose (VBG) levels were lower than 200mg/dL. The Discovery 710 PET/CT (GE Healthcare, Milwaukee, WI, USA) was utilized to scan all 74 patients. FDG PET/CT image acquisition was conducted approximately 1 h after intravenous drop infusion of ^18^F-FDG with a dosage of about 3.7 MBq/kg. All patients were scanned ranging from the parietal skull to the upper femur. The scanning time was 2–3 min/beds, and each patient included 7–8 beds. The entire examination time was nearly 20 min. CT data were used for attenuation correction. The image reconstruction was performed by the ordered-subsets expectation maximization (OSEM) iterative method. Finally, the whole-body PET, CT and PET/CT fusion images were obtained. All PET/CT imaging results were read and confirmed together by two experienced physicians in nuclear medicine at the advanced workstation (AW4.6; GE Medical Systems, Waukesha, WI, USA). The region of interest (ROI) within the cervical tumor was outlined by the percentile threshold method (taking 40% of the maximum standard uptake value (SUVmax) as the threshold). The software automatically generated SUVmax, average standard uptake value (SUVmean), tumor metabolic volume (MTV) and total lesion glycolysis (TLG).

### 2.3. Immunohistochemical Analysis

The rabbit monoclonal (28-8) antibody to PD-L1 (Lot number: ab205921, Abcam, Cambridge, UK) and the rabbit monoclonal antibody to HIF-1α (Lot number: ab51608, Abcam, Cambridge, UK) were used to test the expression of the tumor specimens among these cervical cancer patients. The tumor tissues were fixed using formalin, embedded in paraffin and then sectioned into 4 μm-thick slices by the semi-automatic pathological microtome (craftek, Jinhua, China). Immunohistochemical analyses were performed by a BOND-III Fully Automated IHC Staining System (Leica Biosystems, Wetzlar, Germany). Two independent physicians specializing in pathology were assigned to blindly score the immunostained tissue sections. PD-L1 status was presented by CPS, which was defined as the percentage of PD-L1-positive tumor cells and tumor-associated immune infiltrating cells in all tumor cells, and the results were expressed by a value of 0–100. CPS greater than or equal to 1 was considered positive. Histochemistry score (H-scores) was obtained by the semi-quantitative analysis of the percentage of positive cells and the staining intensity in each section, which was used to reflect HIF-1α expression. The staining intensity score was categorized as 0 (no staining), 1 (light yellow), 2 (yellowish brown) and 3 (brown). The positive cell percentage score was classified as 0 (<5%), 1 (5–25%), 2 (26–50%), 3 (50–75%) and 4 (>75%). An H-score for HIF-1α was calculated as the product of the positive cell percentage score and staining intensity score.

### 2.4. Statistical Analysis

GraphPad Prism 8.0 (GraphPad Software, San Diego, CA, USA) and Statistical Product and Service Solutions (SPSS) Statistics 23.0 (IBM, SPSS Inc., Chicago, IL, USA) were used for data analysis. The normally distributed data are presented as the mean ± standard deviation (SD), and abnormally distributed data are presented as the median (interquartile range). The measurement data between groups were compared by independent samples t-tests or Mann–Whitney U tests. The rate data were compared by chi-square test. Spearman correlation analysis was used for relevance analysis between two variables, the Spearman correlation coefficient (rs) can take values from +1 to −1. A positive correlation occurs when the correlation rs is greater than 0; a negative correlation occurs when the rs is less than. When rs = 0, two variables are irrelevant; when 0 < rs < 1, the closer the rs is to 1, the higher the positive correlation; when −1 < rs < 0, the closer the rs is to −1, the higher the negative correlation. An rs between 0 and 0.3 (or 0 and −0.3) indicates a weak relationship between the two variables. An rs between 0.4 and 0.6 (or −0.4 and −0.6) indicates a moderate relationship between the two variables. An rs between 0.7 and 1 (or −0.7 and −1) indicates a strong relationship between the two variables. Binary logistic regression was used for multivariable analysis. An odds ratio (OR) > 1 indicates that the probability of PD-L1 positivity increases due to the variable; an OR < 1 indicates that the probability of PD-L1 positivity is reduced due to the variable. The 95% confidence interval (CI) of OR should also be calculated. If the 95% CI contains 1, it generally indicates that this factor is meaningless. Receiver operating characteristic (ROC) curves were plotted to detect the possibility of ^18^F-FDG PET/CT semi-quantitative parameters to predict PD-L1 expression. A high accuracy occurs when the area under curve (AUC) > 0.9; a moderate accuracy occurs when 0.7 < AUC ≤ 0.9; a low accuracy occurs when 0.5 < AUC ≤ 0.7; a chance result occurs when the area under curve AUC = 0.5. *p* < 0.05 indicates a statistically significant difference.

## 3. Results

### 3.1. Patients’ Characteristics

The 74 patients’ clinicopathological features and ^18^F-FDG PET/CT metabolic parameters are shown in Table 1. By using immunohistochemical analysis, PD-L1 status was evaluated. Among 74 including cases, 51 (68.9%) were PD-L1 positive and 23 (31.1%) were PD-L1 negative. The median age of the study population was 54.5 years (interquartile range 20.3). Of the 74 patients, 52 (70.3%) had squamous cell carcinoma and the remaining 22 (29.7%) had adenocarcinoma. The number of patients with moderate–well and poor differentiation were 25 (33.8%) and 49 (66.2%); the number of patients with stage I–II and stage III–IV were 40 (54.1%) and 34 (45.9%), respectively. The median primary tumor size was 4.4 cm (interquartile range 2.0 cm). The average SUVmax on ^18^F-FDG PET/CT imaging for the primary tumors was 14.9 (standard deviation 6.9). The mean SUVmean of the primary tumors was 8.8 (standard deviation 3.8). The median TLG of the primary tumors was 154.9 (interquartile range 203.8). The median MTV of the primary tumors was 17.0 (interquartile range 27.4).

### 3.2. Univariate Analysis of the Relationship between PD-L1 Status and Clinicopathologic Characteristics

**Figure 1:**
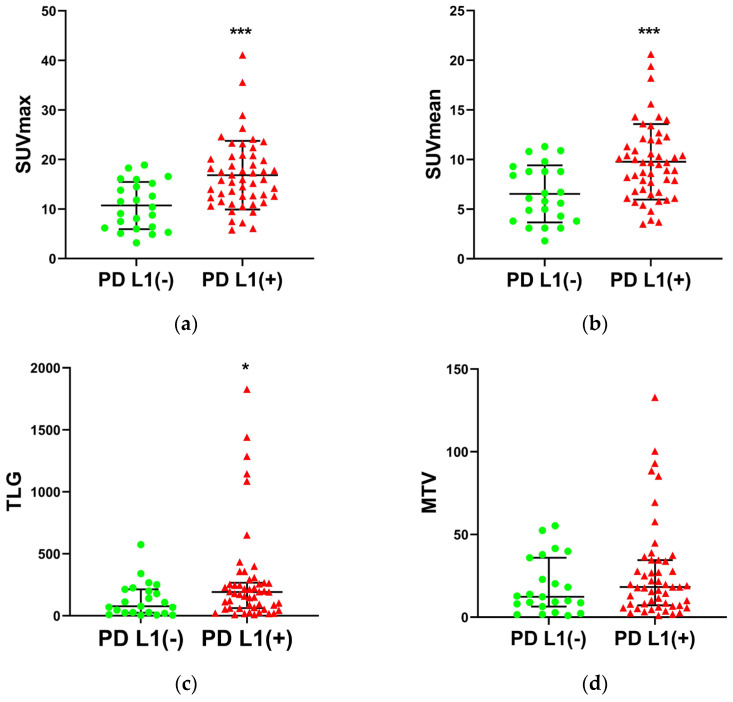
Comparison of SUVmax (**a**), SUVmean (**b**), TLG (**c**) and MTV (**d**) with PD-L1 status (* *p* < 0.05, *** *p* < 0.001).

The SUVmax was significantly higher in PD-L1-positive tumors than in PD-L1-negative tumors (Figure 1a and Table 1, 16.8 ± 6.9 and 10.7 ± 4.8, respectively; *p* = 0.0003), and so was SUVmean (Figure 1b and Table 1, 9.8 ± 3.8 and 6.5 ± 2.9, respectively; *p* = 0.0006). The TLG of PD-L1-positive tumors (191.2 (205.3)) was significantly higher than that of PD-L1-negative tumors (76.4 (189.0)) (Figure 1c and Table 1; *p* = 0.0331). In Figure 1c,d, the TLG and MTV in five cases were dramatically higher than those in the other cases. These five cases all had the characteristics of large primary tumor volume, late T stage and PD-L1-positive expression. The data displayed abnormal distribution and the Mann–Whitney *U* tests used to analyze these data were reliable. The positive rate of PD-L1 in poor differentiation patients (79.6%) was significantly higher than that in moderate–well differentiation patients (48.0%) (Table 1; *p* = 0.0081). The primary tumor size of PD-L1-positive patients (4.5 cm (2.2 cm)) was significantly larger than that of PD-L1-negative patients (4.0 cm (2.0 cm)) (Table 1; *p* = 0.0059).

**Figure 2:**
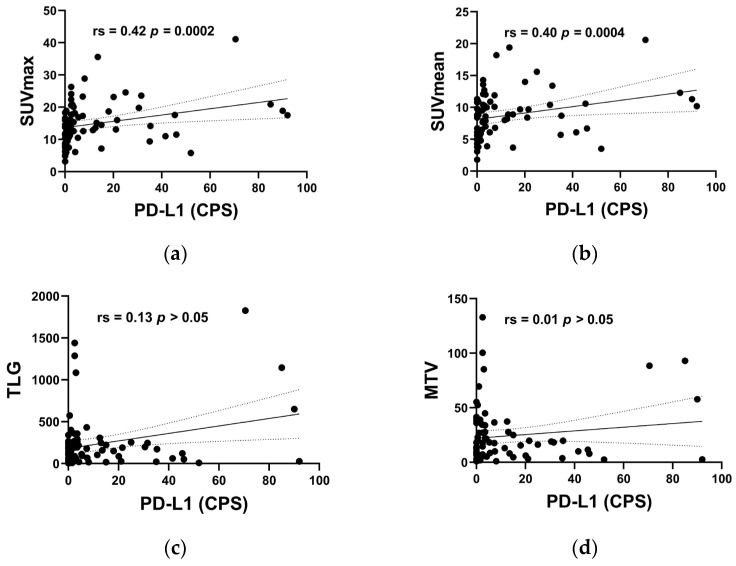
Spearman correlation analysis of SUVmax (**a**), SUVmean (**b**), TLG (**c**) and MTV (**d**) with PD-L1 CPS.

The relevance between primary tumor ^18^F-FDG PET/CT metabolic parameters and PD-L1 CPS were further analyzed by Spearman correlation analysis. As shown in Figure 2a,b, the Spearman rs values to reach statistical significance for SUVmax and SUVmean were 0.42 (*p* = 0.0002) and 0.40 (*p* = 0.0004), respectively. However, the Spearman rs values for TLG and MTV were 0.13 (Figure 2c, *p* > 0.05) and 0.01 (Figure 2d, *p* > 0.05), with no statistical significance. Thus, we concluded that SUVmax and SUVmean exhibit a moderate positive correlation with PD-L1 status in Spearman correlation analysis. These results pointed out that SUVmax and SUVmean were more closely related to the expression of PD-L1.

**Figure 3:**
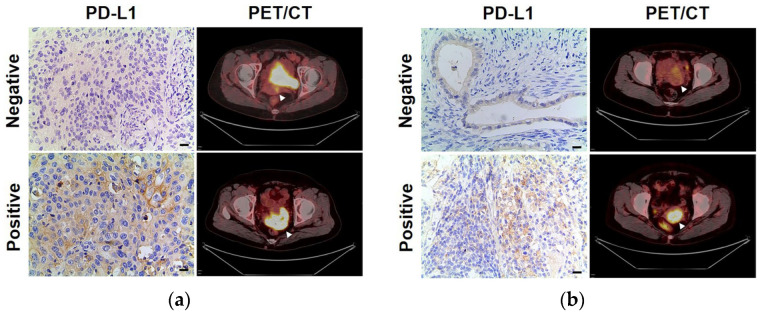
(**a**) Representative imaging of immunohistochemical staining with PD-L1 expression and ^18^F-FDG PET from cervical squamous cell carcinoma patients: a PD-L1-negative patient (upper row) whose primary tumor (arrow) SUVmax, SUVmean, TLG and MTV were 5.3, 3.6, 6.6 and 1.8, respectively, and a PD-L1-positive patient (lower row) whose primary tumor (arrow) SUVmax, SUVmean, TLG and MTV were 18.9, 11.3, 651.6 and 57.8, respectively. (**b**) Representative imaging of immunohistochemical staining with PD-L1 expression and ^18^F-FDG PET from cervical adenocarcinoma patients: a PD-L1-negative patient (upper row) whose primary (arrow) tumor SUVmax, SUVmean, TLG and MTV were 5.1, 3.1, 27.5 and 9.0, respectively, and a PD-L1-positive patient (lower row) whose primary tumor (arrow) SUVmax, SUVmean, TLG and MTV were 9.7, 16.0, 191.2 and 19.8, respectively. Scale bar = 50 μm.

Representative PD-L1 and PET/CT images of patients with cervical squamous cell carcinoma are shown in Figure 3a. The upper row shows a PD-L1-negative patient whose primary tumor SUVmax, SUVmean, TLG and MTV were 5.3, 3.6, 6.6 and 1.8, respectively, and the lower row shows a PD-L1-positive patient whose primary tumor SUVmax, SUVmean, TLG and MTV were 18.9, 11.3, 651.6 and 57.8, respectively. Representative PD-L1 and PET/CT images of patients with cervical adenocarcinoma are shown in Figure 3b, the upper row shows a PD-L1-negative patient whose primary tumor SUVmax, SUVmean, TLG and MTV were 5.1, 3.1, 27.5 and 9.0, respectively, and the lower row shows a PD-L1-positive patient whose primary tumor SUVmax, SUVmean, TLG and MTV were 9.7, 16.0, 191.2 and 19.8, respectively.

No significant difference in age, histologic type, postoperative FIGO stage and MTV were observed between the PD-L1-negative and PD-L1-positive groups (Table 1).

### 3.3. Multivariable Analysis of the Relationship between PD-L1 Status and Clinicopathologic Characteristics

We further used the method of multivariable logistic regression analysis to identify independent prediction factors of PD-L1 status. As shown in Table 2, only the SUVmax of primary tumors (odds ratio, 2.849; 95% CI, 1.066–7.615; *p* = 0.037) and tumor differentiation (odds ratio, 0.168; 95% CI, 0.040–0.703; *p* = 0.015) remained significantly associated with PD-L1 status in the binary multivariable logistic regression analysis. After excluding many confounding factors, these results from multivariable analysis showed that primary tumors with higher SUVmax values and lower degrees of differentiation were more likely to be PD-L1 positive.

### 3.4. ROC Curve Analysis of the Predicting Role of PD-L1 Status by Metabolic Parameters

**Figure 4:**
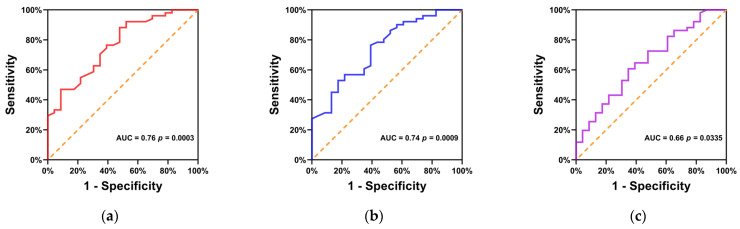
ROC curve analysis of SUVmax (**a**), SUVmean (**b**) and TLG (**c**) for predicting PD-L1 status.

The predicting role of PD-L1 status by ^18^F-FDG PET/CT metabolic parameters including SUVmax, SUVmean and TLG was evaluated by ROC curve analysis. The ROC curve analysis of SUVmax (Figure 4a and Table 3) demonstrated that the AUC was 0.76 (95% CI, 0.65–0.88; *p* = 0.0003), indicating that a diagnosis of PD-L1 status could be predicted by SUVmax with a moderate accuracy. The highest accuracy (77.0%) for predicting PD-L1 expression was obtained with a cut-off value of 10.45. The sensitivity and specificity for predicting PD-L1 expression by SUVmax at 10.45 were 88.2% (95% CI, 76.6–94.5%) and 52.2% (95% CI, 33.0–70.8%), respectively.

The ROC curve analysis of SUVmean (Figure 4b and Table 3) showed that the AUC was 0.74 (95% CI, 0.62–0.86; *p* = 0.0009), indicating that a diagnosis of PD-L1 status could be predicted by SUVmean with a moderate accuracy. The highest accuracy (71.6%) for predicting PD-L1 expression was obtained with a cut-off value of 6.75. The sensitivity and specificity for predicting PD-L1 expression by SUVmean at 6.75 were 76.5% (95% CI, 63.2–86.0%) and 60.9% (95% CI, 40.8–77.8%), respectively.

Similarly, the ROC curve analysis of TLG (Figure 4c and Table 3) showed that the AUC was 0.66 (95% CI, 0.52–0.79; *p* = 0.0335), indicating that a diagnosis of PD-L1 status could be predicted by TLG with a low accuracy. The highest accuracy (62.2%) for predicting PD-L1 expression was obtained with a cut-off value of 143.4. The sensitivity and specificity for predicting PD-L1 expression by TLG at 143.4 were 60.8% (95% CI, 47.1–73.0%) and 65.2% (95% CI, 44.9–81.2%), respectively.

### 3.5. Comprehensive Predictive Ability of Clinicopathological and Metabolic Parameters to PD-L1 Expression

The above results show that SUVmax and tumor differentiation were independent predictors of PD-L1. To evaluate the comprehensive predictive ability of clinicopathological and metabolic parameters for PD-L1 expression, we divided all patients into three groups with graded PD-L1 positive probability according to SUVmax and tumor differentiation. Eight patients with SUVmax < 10.45 and moderate–well differentiation were involved in the low probability group. A total of 27 patients with SUVmax < 10.45 and poor differentiation or SUVmax > 10.45 and moderate–well differentiation were involved in the moderate probability group. A total of 39 patients with SUVmax > 10.45 and poor differentiation were involved in the high probability group. As shown in Table 4, all 8 patients in the low probability group were PD-L1 negative. The PD-L1 pathological positive rates in the moderate and high probability groups were 66.7% and 84.6%, respectively. The difference had statistical significance, *p* < 0.0001.

### 3.6. The Relationship between PD-L1 STATUS and HIF-1α Expression

**Figure 5:**
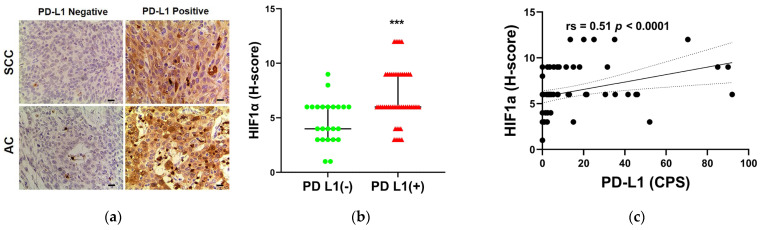
(**a**) Representative imaging of immunohistochemical staining with HIF-1α expression in PD-L1-negative and PD-L1-positive cervical squamous cell carcinoma tissues (up row) and cervical adenocarcinoma tissues (down row). Scale bar = 50 μm. (**b**) Comparison of HIF-1α expression with PD-L1 status (*** *p* < 0.001). (**c**) Spearman correlation analysis of HIF-1α expression with PD-L1 CPS.

In order to evaluate the internal mechanism that FDG uptake was closely related to the expression of PD-L1, we performed HIF1α staining and quantitative analysis on cervical cancer sections. The upper row of Figure 5a shows representative sections of low and high HIF-1α expression in PD-L1-negative and PD-L1-positive cervical squamous cell carcinoma tissues. The lower row of Figure 5a shows representative sections of low and high HIF-1α expression in PD-L1-negative and PD-L1-positive cervical adenocarcinoma tissues. The HIF-1α H-score of PD-L1-positive tumors was significantly higher than that of PD-L1-negative tumors (Figure 5b; *p* = 0.0004). The H-score of HIF-1α was moderately positively correlated with PD-L1 CPS by Spearman correlation analysis (Figure 5c, rs = 0.51; *p* < 0.0001). According to these findings, HIF-1α activation may be the potential mechanism of high FDG uptake in PD-L1-positive tumors.

## 4. Discussion

Immune checkpoint agents targeting the PD-1/PD-L1 signaling pathway have been successfully applied to the treatment of different types of malignant tumors, including non-small-cell lung cancer [18], kidney cancer [19], bladder cancer [20] and malignant melanoma [21]. The response to anti-PD-1/PD-L1 treatment could be predicted by expression of PD-L1 within a tumor. Pembrolizumab, a PD-1 inhibitor, has been approved for treatment in persistent, recurrent or metastatic cervical cancer patients with PD-L1-positive tumors (CPS ≥ 1). Immunohistochemical analysis is the standard method to evaluate tumor PD-L1 expression, nevertheless, this method always requires tumor tissues which are invasively obtained by hysteroscopy or surgical resection. Therefore, ^18^F-FDG PET/CT imaging, which is broadly applied in the diagnosis, staging, follow-up and recurrence monitoring of malignant tumors, may be an alternative non-invasive strategy for predicting PD-L1 expression within a tumor; this information would be of great value for blocking PD-1/PD-L1 immunotherapy. In several human cancers [22], PD-L1 expression has been recently reported to be positively correlated with the uptake of ^18^F-FDG.

In previous studies, SUVmax has almost been the primary predictor of PD-L1 expression in several malignant tumors. In ovarian cancer [23], non-small cell lung cancer (NSCLC) [24], gastric cancer [25], bladder cancer [15] and nasopharyngeal carcinoma [26] the SUVmax cut-off values were 10.5, 12.5, 8.55, 22.7 and 6.7, respectively. Correspondingly, the highest accuracy for predicting the PD-L1 status was 61.8%, 80.7%, 67.2%, 77.8% and 78.6%. Moreover, Liang Zhao et al. have also reported that a TLG cut-off value of 41.3 obtained the highest accuracy of 71.4% for predicting PD-L1 expression in nasopharyngeal carcinoma. In our study, the predicting role of PD-L1 status by metabolic parameters was evaluated by ROC curve analysis. When the cut-off values of SUVmax, SUVmean and TLG were 10.45, 6.75 and 143.4, respectively, the highest accuracy for predicting PD-L1 expression was 77.0%, 71.6% and 62.2%, respectively. Consistent with the above literature, our research concluded that SUVmax was an optimal predictor of PD-L1 status.

The cervical cancer tissue microarray sample immunohistochemistry analysis performed by Saglam et al. showed that PD-L1 expression was higher in poorly differentiated tumors than in moderately differentiated tumors [27]. The expression of PD-L1 was usually higher in poorly differentiated tumors; this finding has been reported for several other cancers, such as non-small cell lung carcinoma [28,29], thyroid cancer [30] and gallbladder cancer [31]. In our study, the degree of tumor differentiation was a negative independent predictor for PD-L1 status in the binary multivariate logistic regression analysis. The assessment of the comprehensive predictive ability of PD-L1 expression indicated that combining SUVmax with tumor differentiation exhibits an effective prediction function for PD-L1 expression.

As early as 2015, Chang et al. reported the role for PD-L1 in influencing tumor cell metabolism [32]. They found that inhibiting the AKT/mTOR signaling pathway by blocking tumor PD-L1 can reduce expression of glycolysis enzymes, suggesting there is an important role for PD-L1 in tumor glucose utilization. It was also demonstrated that high PD-L1 expression could promote the glycolysis of acute myeloid leukemia cells through the Akt/mTOR signaling transduction pathway [33]. In addition, Wang et al. [34] found that the integrin β4/SNAI1/SIRT3 signaling pathway could be activated by PD-L1, which triggered the up-regulation of glucose metabolism in human cervical cancer cell lines. It has been reported by some previous researchers that HIF-1α plays an important role in triggering the glycolysis of tumor cells and enhancing their ^18^F-FDG uptake [35,36,37]. In addition, HIF-1α could upregulate PD-L1 expression by binding directly to the hypoxia-responsive element (HRE) in the PD-L1 promoter [38,39,40]. According to these findings, the positive correlation between PD-L1 expression and ^18^F-FDG accumulation could be mostly resulting from the activation of HIF-1α pathway. Our findings suggested that PD-L1 expression was correlated with glucose metabolism accumulation, which means ^18^F-FDG PET/CT may be of value for predicting tumor PD-L1 expression in cervical cancer patients. However, the possible underlying molecular mechanism of the correlation between PD-L1 expression and ^18^F-FDG accumulation has not yet been fully revealed. Our current research results confirmed this hypothesis, the HIF-1α expression of PD-L1-positive tumors was found to be higher than that of PD-L1-negative tumors. Additionally, there was a positive correlation between the H-score of HIF-1α and PD-L1 CPS in our current study.

There are several limitations in our present study. Firstly, this was a single-institution and retrospective study with a relatively small patient population. Therefore, there is a necessity to carry out a multicenter study with a larger sample size for determining the cut-off values for the ^18^F-FDG metabolic parameters in future. Secondly, this study did not enroll patients who were treated with PD-1/PD-L1 blockades, it is still unclear whether there is a role for ^18^F-FDG uptake in predicting the therapeutic efficacy and prognosis of immunotherapy. Finally, although ^18^F-FDG PET/CT has shown a moderate predictive value, the pathologic biopsy will not be replaced in routine clinical work for assessing the status of PD-L1. Despite all the above, the present study will contribute to promoting the development of other non-invasive methods aimed at the prediction of PD-L1 expression. Exploration of immune PET with PD1/PD-L1-targeted agents would supply another potential research direction in the near future.

## 5. Conclusions

Our results indicated that ^18^F-FDG uptake by cervical cancer was closely related to PD-L1 expression and could be of usefulness for predicting PD-L1 status. The optimum predictors were SUVmax and tumor differentiation. The high FDG uptake in PD-L1-positive tumors might be related to HIF-1α activation.

## Figures and Tables

**Table 1:** Univariate analysis of the relationship between PD-L1 status and clinicopathologic characteristics.

Variable	All	PD-L1 Negative	PD-L1 Positive	*p*-Value
Number of patients	74	23	51	
Age, year; median (IQR)	54.5 (20.3)	54.0 (23.0)	56.0 (18.0)	0.7962
<55	37	12	25	0.8017
≥55	37	11	26	
Histologic type				
SCC	52	14	38	0.2348
AC	22	9	13	
Tumor differentiation				
Moderate–Well	25	13	12	0.0081
Poor	49	10	39	
FIGO stage *				
I–II	40	12	28	0.8275
III–IV	34	11	23	
Tumor size, cm; median (IQR)	4.4 (2.0)	4.0 (2.0)	4.5 (2.2)	0.0059
SUVmax, mean ± SD	14.9 ± 6.9	10.7 ± 4.8	16.8 ± 6.9	0.0003
SUVmean, mean ± SD	8.8 ± 3.8	6.5 ± 2.9	9.8 ± 3.8	0.0006
TLG, median (IQR)	154.9 (203.8)	76.4 (189.0)	191.2 (205.3)	0.0331
MTV, median (IQR)	17.0 (27.4)	12.4 (33.1)	18.3 (27.3)	0.3078

IQR: interquartile range, SCC: squamous cell carcinoma, AC: adenocarcinoma, SUV: standardized uptake value, SD: standard deviation, TLG: total lesion glycolysis, MTV: metabolic tumor volume, * 2018 FIGO staging used (postoperative).

**Table 2:** Multivariable analysis of the relationship between PD-L1 status and clinicopathologic characteristics.

Variable	Odds Ratio	95% CI	*p*-Value
SUVmax	2.849	1.066–7.615	0.037
Tumor differentiation	0.168	0.040–0.703	0.015

SUV: standardized uptake value, CI: confidence interval.

**Table 3:** ROC curve analysis of the predicting role of PD-L1 status by metabolic parameters.

	SUVmax	SUVmean	TLG
AUC (95% CI)	0.76 (0.65–0.88)	0.74 (0.62–0.86)	0.66 (0.52–0.79)
*p* value	0.0003	0.0009	0.0335
cut-off value	10.45	6.75	143.4
Sensitivity (95% CI)	88.2% (76.6–94.5%)	76.5% (63.2–86.0%)	60.8% (47.1–73.0%)
Specificity (95% CI)	52.2% (33.0–70.8%)	60.9% (40.8–77.8%)	65.2% (44.9–81.2%)
Accuracy	77.0%	71.6%	62.2%

SUV: standardized uptake value, AUC: area under the curve, CI: confidence interval.

**Table 4:** PD-L1 expression in the low-, moderate-, and high-potential groups.

Probability	Number of Patients	PD-L1 Negative	PD-L1 Positive	*p*-Value
Low	8	8 (100%)	0 (0%)	*p* < 0.0001
Moderate	27	9 (33.3%)	18 (66.7%)	
High	39	6 (15.4%)	33 (84.6%)	

## Data Availability

The data presented in this study are available on reasonable request from the corresponding author.

## References

[1] Sung H., Ferlay J., Siegel R.L., Laversanne M., Soerjomataram I., Jemal A., Bray F. (2021). Global Cancer Statistics 2020: GLOBOCAN Estimates of Incidence and Mortality Worldwide for 36 Cancers in 185 Countries. CA A Cancer J. Clin..

[2] Wang Y., Li G. (2019). PD-1/PD-L1 blockade in cervical cancer: Current studies and perspectives. Front. Med..

[3] Liu M.C., Tewari K.S. (2022). Current and emerging immunotherapies for recurrent cervical cancer. Clin. Adv. Hematol. Oncol. HO.

[4] Hegde P.S., Chen D.S. (2020). Top 10 Challenges in Cancer Immunotherapy. Immunity.

[5] Chung H.C., Ros W., Delord J.P., Perets R., Italiano A., Shapira-Frommer R., Manzuk L., Piha-Paul S.A., Xu L., Zeigenfuss S. (2019). Efficacy and Safety of Pembrolizumab in Previously Treated Advanced Cervical Cancer: Results from the Phase II KEYNOTE-158 Study. J. Clin. Oncol..

[6] Colombo N., Dubot C., Lorusso D., Caceres M.V., Hasegawa K., Shapira-Frommer R., Tewari K.S., Salman P., Hoyos Usta E., Yañez E. (2021). Pembrolizumab for Persistent, Recurrent, or Metastatic Cervical Cancer. N. Engl. J. Med..

[7] Salem A.E., Shah H.R., Covington M.F., Koppula B.R., Fine G.C., Wiggins R.H., Hoffman J.M., Morton K.A. (2022). PET-CT in Clinical Adult Oncology: I. Hematologic Malignancies. Cancers.

[8] Covington M.F., Koppula B.R., Fine G.C., Salem A.E., Wiggins R.H., Hoffman J.M., Morton K.A. (2022). PET-CT in Clinical Adult Oncology: II. Primary Thoracic and Breast Malignancies. Cancers.

[9] Koppula B.R., Fine G.C., Salem A.E., Covington M.F., Wiggins R.H., Hoffman J.M., Morton K.A. (2022). PET-CT in Clinical Adult Oncology: III. Gastrointestinal Malignancies. Cancers.

[10] Salem A.E., Fine G.C., Covington M.F., Koppula B.R., Wiggins R.H., Hoffman J.M., Morton K.A. (2022). PET-CT in Clinical Adult Oncology-IV. Gynecologic and Genitourinary Malignancies. Cancers.

[11] Wiggins R.H., Hoffman J.M., Fine G.C., Covington M.F., Salem A.E., Koppula B.R., Morton K.A. (2022). PET-CT in Clinical Adult Oncology-V. Head and Neck and Neuro Oncology. Cancers.

[12] Fine G.C., Covington M.F., Koppula B.R., Salem A.E., Wiggins R.H., Hoffman J.M., Morton K.A. (2022). PET-CT in Clinical Adult Oncology-VI. Primary Cutaneous Cancer, Sarcomas and Neuroendocrine Tumors. Cancers.

[13] Zhang L.F., Li J.L., Wang Y.H., Tai X.H., Liu L., Zhang X.X., An Y.W., Li H.L. (2021). The Correlation Between (18)F-Fluorodeoxyglucose-Positron Emission Tomography/Computed Tomography Semiquantitative Parameters and the Clinical Features and Pathological Biological Indexes of Gastric Cancer. Cancer Biother. Radiopharm..

[14] Lv Z., Fan J., Xu J., Wu F., Huang Q., Guo M., Liao T., Liu S., Lan X., Liao S. (2018). Value of (18)F-FDG PET/CT for predicting EGFR mutations and positive ALK expression in patients with non-small cell lung cancer: A retrospective analysis of 849 Chinese patients. Eur. J. Nucl. Med. Mol. Imaging.

[15] Chen R., Zhou X., Liu J., Huang G. (2019). Relationship between the expression of PD-1/PD-L1 and (18)F-FDG uptake in bladder cancer. Eur. J. Nucl. Med. Mol. Imaging.

[16] Hu B., Chen W., Zhang Y., Shi H., Cheng D., Xiu Y. (2020). (18)F-FDG maximum standard uptake value predicts PD-L1 expression on tumor cells or tumor-infiltrating immune cells in non-small cell lung cancer. Ann. Nucl. Med..

[17] Hirakata T., Fujii T., Kurozumi S., Katayama A., Honda C., Yanai K., Tokuda S., Nakazawa Y., Obayashi S., Yajima R. (2020). FDG uptake reflects breast cancer immunological features: The PD-L1 expression and degree of TILs in primary breast cancer. Breast Cancer Res. Treat..

[18] O’Brien M., Paz-Ares L., Marreaud S., Dafni U., Oselin K., Havel L., Esteban E., Isla D., Martinez-Marti A., Faehling M. (2022). Pembrolizumab versus placebo as adjuvant therapy for completely resected stage IB-IIIA non-small-cell lung cancer (PEARLS/KEYNOTE-091): An interim analysis of a randomised, triple-blind, phase 3 trial. Lancet. Oncol..

[19] Voss M.H., Azad A.A., Hansen A.R., Gray J.E., Welsh S.J., Song X., Kuziora M., Meinecke L., Blando J., Achour I. (2022). A Randomized Phase II Study of MEDI0680 in Combination with Durvalumab versus Nivolumab Monotherapy in Patients with Advanced or Metastatic Clear-cell Renal Cell Carcinoma. Clin. Cancer Res..

[20] Basile G., Bandini M., Gibb E.A., Ross J.S., Raggi D., Marandino L., Costa de Padua T., Crupi E., Colombo R., Colecchia M. (2022). Neoadjuvant Pembrolizumab and Radical Cystectomy in Patients with Muscle-Invasive Urothelial Bladder Cancer: 3-Year Median Follow-Up Update of PURE-01 Trial. Clin. Cancer Res..

[21] Long G.V., Luke J.J., Khattak M.A., de la Cruz Merino L., Del Vecchio M., Rutkowski P., Spagnolo F., Mackiewicz J., Chiarion-Sileni V., Kirkwood J.M. (2022). Pembrolizumab versus placebo as adjuvant therapy in resected stage IIB or IIC melanoma (KEYNOTE-716): Distant metastasis-free survival results of a multicentre, double-blind, randomised, phase 3 trial. Lancet. Oncol..

[22] Kaira K., Kuji I., Kagamu H. (2021). Value of (18)F-FDG-PET to predict PD-L1 expression and outcomes of PD-1 inhibition therapy in human cancers. Cancer Imaging Off. Publ. Int. Cancer Imaging Soc..

[23] Choi Y.J., Jo K., Hwang S.H., Jeong Y., Lee J.Y., Kim S., Kim S.W., Kim Y.T., Kang W.J. (2021). Association between PD-L1 expression and (18)F-FDG uptake in ovarian cancer. Ann. Nucl. Med..

[24] Wu X., Huang Y., Zhao Q., Wang L., Song X., Li Y., Jiang L. (2020). PD-L1 expression correlation with metabolic parameters of FDG PET/CT and clinicopathological characteristics in non-small cell lung cancer. EJNMMI Res..

[25] Chen R., Chen Y., Huang G., Liu J. (2019). Relationship between PD-L1 expression and (18)F-FDG uptake in gastric cancer. Aging.

[26] Zhao L., Zhuang Y., Fu K., Chen P., Wang Y., Zhuo J., Liao X., Chen H., Lin Q. (2020). Usefulness of [(18)F]fluorodeoxyglucose PET/CT for evaluating the PD-L1 status in nasopharyngeal carcinoma. Eur. J. Nucl. Med. Mol. Imaging.

[27] Saglam O., Zhou J., Wang X., Conejo-Garcia J.R. (2020). PD-L1 Expression Correlates with Young Age and CD8+ TIL Density in Poorly Differentiated Cervical Squamous Cell Carcinoma. Int. J. Gynecol. Pathol..

[28] Blichárová A., Tancoš V., Benetinová Z., Verbóová Ľ., Grendár M., Mazuráková A., Plank L., Mechírová E. (2023). Programmed death ligand-1 expression and its association with the degree of differentiation and the presence of necrosis in non-small cell lung carcinoma. Pathol. Res. Pract..

[29] Akhave N., Zhang J., Bayley E., Frank M., Chiou S.H., Behrens C., Chen R., Hu X., Parra E.R., Lee W.C. (2022). Immunogenomic profiling of lung adenocarcinoma reveals poorly differentiated tumors are associated with an immunogenic tumor microenvironment. Lung Cancer.

[30] Cameselle-García S., Abdulkader-Sande S., Sánchez-Ares M., Rodríguez-Carnero G., Garcia-Gómez J., Gude-Sampedro F., Abdulkader-Nallib I., Cameselle-Teijeiro J.M. (2021). PD-L1 expression and immune cells in anaplastic carcinoma and poorly differentiated carcinoma of the human thyroid gland: A retrospective study. Oncol. Lett..

[31] Albrecht T., Brinkmann F., Albrecht M., Lonsdorf A.S., Mehrabi A., Hoffmann K., Kulu Y., Charbel A., Vogel M.N., Rupp C. (2021). Programmed Death Ligand-1 (PD-L1) Is an Independent Negative Prognosticator in Western-World Gallbladder Cancer. Cancers.

[32] Chang C.H., Qiu J., O’Sullivan D., Buck M.D., Noguchi T., Curtis J.D., Chen Q., Gindin M., Gubin M.M., van der Windt G.J. (2015). Metabolic Competition in the Tumor Microenvironment Is a Driver of Cancer Progression. Cell.

[33] Ma P., Xing M., Han L., Gan S., Ma J., Wu F., Huang Y., Chen Y., Tian W., An C. (2020). High PD-L1 expression drives glycolysis via an Akt/mTOR/HIF-1α axis in acute myeloid leukemia. Oncol. Rep..

[34] Wang S., Li J., Xie J., Liu F., Duan Y., Wu Y., Huang S., He X., Wang Z., Wu X. (2018). Programmed death ligand 1 promotes lymph node metastasis and glucose metabolism in cervical cancer by activating integrin β4/SNAI1/SIRT3 signaling pathway. Oncogene.

[35] Pal S., Sharma A., Mathew S.P., Jaganathan B.G. (2022). Targeting cancer-specific metabolic pathways for developing novel cancer therapeutics. Front. Immunol..

[36] Li Y., Zhao L., Huo Y., Yang X., Li Y., Xu H., Li X.F. (2022). Visualization of hypoxia in cancer cells from effusions in animals and cancer patients. Front. Oncol..

[37] Elzakra N., Kim Y. (2021). HIF-1α Metabolic Pathways in Human Cancer. Adv. Exp. Med. Biol..

[38] Chen Z.Q., Zuo X.L., Cai J., Zhang Y., Han G.Y., Zhang L., Ding W.Z., Wu J.D., Wang X.H. (2023). Hypoxia-associated circPRDM4 promotes immune escape via HIF-1α regulation of PD-L1 in hepatocellular carcinoma. Exp. Hematol. Oncol..

[39] Yong J., Gröger S., von Bremen J., Meyle J., Ruf S. (2022). Immunorthodontics: PD-L1, a Novel Immunomodulator in Cementoblasts, Is Regulated by HIF-1α under Hypoxia. Cells.

[40] Song S., Zhang Y., Duan X., Liu C., Du Y., Wang X., Luo Y., Cui Y. (2023). HIF-1α/IL-8 axis in hypoxic macrophages promotes esophageal cancer progression by enhancing PD-L1 expression. Cancer Gene Ther..

